# Supplementary material for: An open-source framework for large-scale, flexible evaluation of biomedical text mining systems
Source: J Biomed Discov Collab. 2008 Jan 29;3:1. doi: 10.1186/1747-5333-3-1 (PMC2276192; doi:10.1186/1747-5333-3-1)
Supplement: Additional file 1 — Evaluation platform source code. The additional file contains the software evaluation framework discussed in this paper. The most current release can be downloaded from SourceForge.net [1]. See the accompanying README file for instructions on installation and use. Also included in the distribution is a collection of UIMA wrappers for some commonly used BioNLP tools and annotated corpora. [file 1747-5333-3-1-S1.zip › bionlp_uima_repository-1.0.1/docs/api/edu/uchsc/ccp/util/nlp/mention/comparison/IdenticalSlotMentionComparator.html]

IdenticalSlotMentionComparator (Center for Computational Pharmacology Util/UIMA API)


|  |  |  |
| --- | --- | --- |
| **Overview** \| **Package** \| **Class** \| **Tree** \| **Deprecated** \| **Index** \| **Help** | |  |
| **PREV CLASS**   **NEXT CLASS** | **FRAMES**    **NO FRAMES**     **All Classes** |  |
| SUMMARY: NESTED \| FIELD \| CONSTR \| METHOD | DETAIL: FIELD \| CONSTR \| METHOD |  |


---


## edu.uchsc.ccp.util.nlp.mention.comparison Class IdenticalSlotMentionComparator

```
java.lang.Object
  edu.uchsc.ccp.util.nlp.mention.comparison.MentionComparator
      edu.uchsc.ccp.util.nlp.mention.comparison.IdenticalMentionComparator
          edu.uchsc.ccp.util.nlp.mention.comparison.IdenticalSlotMentionComparator
```

**All Implemented Interfaces:**: SlotMentionComparator, Comparator<Mention>

---

``` public class IdenticalSlotMentionComparator extends IdenticalMentionComparator implements SlotMentionComparator ```

---

**Field Summary**

**Fields inherited from class edu.uchsc.ccp.util.nlp.mention.comparison.MentionComparator**

`MULTIPLIER`


**Constructor Summary**

`IdenticalSlotMentionComparator()`


| **Method Summary** | |
| --- | --- |
| `int` | `compare(Mention mention1, Mention mention2, SpanComparator spanComparator, int maximumComparisonDepth, int depth)`             Compare two mentions. |
| `int` | `compare(SlotMention slotMention1, SlotMention slotMention2, SpanComparator spanComparator, int maximumComparisonDepth, int depth)`             Compare two SlotMentions. |

**Methods inherited from class edu.uchsc.ccp.util.nlp.mention.comparison.IdenticalMentionComparator**

`hasEquivalentMentionNames`

**Methods inherited from class edu.uchsc.ccp.util.nlp.mention.comparison.MentionComparator**

`compare, equalComplexSlotMentions, equalSlotMentions`

**Methods inherited from class java.lang.Object**

`clone, equals, finalize, getClass, hashCode, notify, notifyAll, toString, wait, wait, wait`

**Methods inherited from interface java.util.Comparator**

`equals`

**Constructor Detail**

### IdenticalSlotMentionComparator

```
public IdenticalSlotMentionComparator()
```


**Method Detail**

### compare

```
public int compare(Mention mention1,
                   Mention mention2,
                   SpanComparator spanComparator,
                   int maximumComparisonDepth,
                   int depth)
```

:   Compare two mentions. They must be SlotMentions or else an UnsupportedOperationException is thrown. In order to be identical (and return 0),
    they must have the name mention name, and the identical slot values

    :   **Specified by:**: `compare` in class `MentionComparator`

---


### compare

```
public int compare(SlotMention slotMention1,
                   SlotMention slotMention2,
                   SpanComparator spanComparator,
                   int maximumComparisonDepth,
                   int depth)
```

:   Compare two SlotMentions. In order to be identical (and return 0), they must have the same mention name, and the identical slot values

    :   **Specified by:**: `compare` in interface `SlotMentionComparator`
    :   **Parameters:**: `slotMention1` -: `slotMention2` -: `maximumComparisonDepth` -: `depth` -: `spanComparator` -: `compareLinkedAnnotationSpans` - **Returns:**


---


|  |  |  |
| --- | --- | --- |
| **Overview** \| **Package** \| **Class** \| **Tree** \| **Deprecated** \| **Index** \| **Help** | |  |
| **PREV CLASS**   **NEXT CLASS** | **FRAMES**    **NO FRAMES**     **All Classes** |  |
| SUMMARY: NESTED \| FIELD \| CONSTR \| METHOD | DETAIL: FIELD \| CONSTR \| METHOD |  |


---
